# Leadership and Teamwork in Trauma and Resuscitation

**DOI:** 10.5811/westjem.2016.7.29812

**Published:** 2016-08-22

**Authors:** Kelsey Ford, Michael Menchine, Elizabeth Burner, Sanjay Arora, Kenji Inaba, Demetrios Demetriades, Bertrand Yersin

**Affiliations:** * Keck School of Medicine of the University of Southern California, Department of Emergency Medicine, Los Angeles, California; † Keck School of Medicine of the University of Southern California, Department of Surgery, Los Angeles, California; ‡ University of Lausanne, Department of Medicine, Lausanne, Switzerland

## Abstract

**Introduction:**

Leadership skills are described by the American College of Surgeons’ Advanced Trauma Life Support (ATLS) course as necessary to provide care for patients during resuscitations. However, leadership is a complex concept, and the tools used to assess the quality of leadership are poorly described, inadequately validated, and infrequently used. Despite its importance, dedicated leadership education is rarely part of physician training programs. The goals of this investigation were the following: 1. Describe how leadership and leadership style affect patient care; 2. Describe how effective leadership is measured; and 3. Describe how to train future physician leaders.

**Methods:**

We searched the PubMed database using the keywords “leadership” and then either “trauma” or “resuscitation” as title search terms, and an expert in emergency medicine and trauma then identified prospective observational and randomized controlled studies measuring leadership and teamwork quality. Study results were categorized as follows: 1) how leadership affects patient care; 2) which tools are available to measure leadership; and 3) methods to train physicians to become better leaders.

**Results:**

We included 16 relevant studies in this review. Overall, these studies showed that strong leadership improves processes of care in trauma resuscitation including speed and completion of the primary and secondary surveys. The optimal style and structure of leadership are influenced by patient characteristics and team composition. Directive leadership is most effective when Injury Severity Score (ISS) is high or teams are inexperienced, while empowering leadership is most effective when ISS is low or teams more experienced. Many scales were employed to measure leadership. The Leader Behavior Description Questionnaire (LBDQ) was the only scale used in more than one study. Seven studies described methods for training leaders. Leadership training programs included didactic teaching followed by simulations. Although programs differed in length, intensity, and training level of participants, all programs demonstrated improved team performance.

**Conclusion:**

Despite the relative paucity of literature on leadership in resuscitations, this review found leadership improves processes of care in trauma and can be enhanced through dedicated training. Future research is needed to validate leadership assessment scales, develop optimal training mechanisms, and demonstrate leadership’s effect on patient-level outcome.

## INTRODUCTION

Coordinating doctors, nurses, and ancillary staff to care for patients requires teamwork and leadership. This is particularly true in emergency settings where providers from numerous specialties converge to care for critically ill patients with limited data and under strict time constraints. The most recent Advanced Trauma Life Support (ATLS) guidelines have codified leadership’s importance by emphasizing that for a team to “perform effectively one team member should assume the role of the team leader.”^1^ However, unlike the majority of other key elements of trauma care (e.g. airway assessment), the ATLS manual does not provide specific teamwork training recommendations or guidelines for leadership. As a result, the leadership and teamwork structure for trauma care is generally dictated by provider preference, institutional history, and local culture rather than uniform standards.

Leadership styles are divided into two main categories: directive or empowering. Directive leadership is typical of a military chain of command. The commanding officer explicitly instructs subordinates on which tasks to perform and when to perform them, effectively managing and supervising the decision-making process through role distribution and flow of information.^2^, ^3^ This type of leadership is effective when tasks are simple, straightforward, and/or the leader is the only team member with expertise.^4^ In empowering leadership, leaders delegate responsibility, allowing colleagues to make decisions while the leader focuses on team communication and coordination. The primacy of directive leadership has been increasingly challenged. Newer theories postulate that empowering (shared) leadership is more effective when tasks are complex.^4^ These theories suggest the more complex a task, the more necessary it is for team members to share the responsibility of management of information, communication, and adaptability to achieve success.^4^

However, the optimal leadership style and team structure for trauma is largely unstudied. Trauma resuscitation has elements that are simple/task-oriented and components that are highly complex requiring team member coordination. As such, directive and empowering leadership styles might both play a role. Furthermore, the development of emergency medicine as a specialty has changed the structure of leadership in trauma. Cross-disciplinary and shared leadership structures now exist in which trauma surgeons and emergency physicians mutually make decisions for the benefit of the patient. Research to elucidate the optimal style and structure of leadership in trauma is limited by a lack of validated tools to measure the quality of leadership and teamwork. Once standards are developed, training programs can be created on the basis of strong scientific evidence.

The goal of this paper was to review the scientific literature on leadership and teamwork in trauma and resuscitation patients. Specifically, we evaluated 1) how leadership and teamwork affect patient care, 2) which tools are available to measure effective leadership or teamwork, and 3) what methods can be used to train physicians to become better team leaders/team members.

## METHODS

We searched the PubMed database using the keywords “leadership” and then either “trauma” or “resuscitation” as title search terms from 1973 through 2014. This resulted in the identification of a total of 345 and 158 abstracts respectively. An expert in emergency medicine and trauma reviewed these abstracts and identified prospective observational and randomized controlled studies measuring leadership and teamwork quality. We included studies on medical resuscitation due to the paucity of studies that specifically addressed leadership in trauma. Due to medical resuscitation’s similar dependence on teamwork under strict time constraints, we believe that these situations parallel sufficiently to extrapolate meaningful data for trauma teams. We excluded studies in which the resuscitation occurred in or was simulated in other patient care settings including the operating room or intensive care unit. Additional exclusion criteria included manuscripts not available in English and manuscripts in which leadership/teamwork were mentioned but not the focus of the paper. After applying our exclusions we had 10 relevant articles focusing on trauma and six additional articles on medical resuscitation. The database search was followed by an ancestral search of the references of included articles using the same inclusion and exclusion criteria. Since very little information exists on this topic, experts were consulted to identify additional relevant manuscripts.

We organized results according to which of the three study questions they addressed: 1. How does leadership/leadership style affect patient care and team performance? 2. How can effective leadership/teamwork be measured? 3. How can leaders be trained?

## RESULTS

After applying our inclusion and exclusion criteria, we included 16 articles in this review. A summary of the selected articles is displayed in two tables: Table 1 includes 10 articles on trauma, and Table 2 includes six articles on medical resuscitation.

### 1. Does leadership affect patient care and team performance?

Strong leadership and teamwork improve processes of care in trauma including completion of primary and secondary surveys while the optimal style and structure of leadership are influenced by patient characteristics and team composition.

Of the 16 studies included in this review, nine examined how leadership and teamwork affect patient care. The literature suggests that effective leadership is associated with better processes of care in both trauma cases and medical resuscitation (Tables 1 and 2). With respect to trauma cases,^4^ studies evaluated how leadership and teamwork affect time to complete ATLS standards. Specifically, Hoff et al. demonstrated that teams with designated trauma team leaders (command physicians) were more likely to adhere to ATLS standards of care. Teams with a leader had higher rates of completion of their secondary survey and formulation of a plan than teams without an identified leader.^5^ In Lubbert et al., a lack of leadership led to more errors and delays in performing expected tasks during the primary and secondary surveys.^6^ In Capella, strong teamwork led to decreased time to computed tomography (CT), endotracheal intubation, and transfer to the operating room.^7^ Similarly, Steinemann showed that leadership and teamwork led to decreased resuscitation time and increased “near-perfect” task completion during the primary survey.^8^

Five of the studies focusing on medical resuscitation showed leadership/teamwork increased successful task completion.^9^–^13^ For example, in Cooper et al., strong leadership led to improvements in task-performance scores that included items like basic ventilation and chest compressions.^9^ In Yeung et al, strong leadership was associated with higher quality and more successful cardiopulmonary resuscitations.^13^ Hunziker authored two studies that noted students trained in leadership had fewer delays in initiating basic life support than students who were trained on technical skills alone.^11^,^12^ Similarly, in Marsch et al. poor leadership and task distribution were associated with poor team performance as measured by longer time to deliver basic life support and perform cardioversion, as well as fewer successful resuscitations.^10^

The optimal style and structure of leadership varied based on patient characteristics and team composition. Directive leadership was shown to be most effective when patients’ Injury Severity Scores (ISS) were high or teams were less experienced, while empowering leadership was more effective when ISS was low or teams were more experienced.^14^ Leaders who actively participated in patient care, for example by performing procedures, had lower team-performance scores because the leader was unable to oversee, monitor and supervise the resuscitation.^9^ The structure of leadership also impacts patient care. When emergency physicians and surgeons share leadership roles by collaborating, there was better decision agreement and faster delivery of care than if each physician made decisions unilaterally or independently (solo or parallel decision-making).^15^ See Supplemental Digital Content (SCD) 1 for diagram of leadership structures.

### 2. How can effective leadership/teamwork be measured?

While there is no consensus on the most effective tool to measure leadership or teamwork, at this time the Leader Behavior Description Questionnaire (LBDQ) has been the most widely used and validated.

Of the 16 studies, eight assessed quality of leadership using standardized scales that focused on various components of leadership/teamwork behavior including communication. Four studies used tools to evaluate team performance that included an evaluation of the team’s leadership as a sub-scale,^6^–^8^, ^16^ while four studies evaluated leadership in isolation.^9^, ^13^, ^15^, ^17^ Lubbert et al used an attending surgeon to evaluate video-recorded traumas over a two-year period using an internally developed leadership/team work scoring scale. The scale focused on errors in team functioning in 10 domains including the following: timing of complete team arrival, organization/communication, protective measures, and patient transfer. The leader was rated on five items: clearly evident, efficient, perform the resuscitation in the correct order, work according to protocol, and maintain the patient under constant supervision.^6^ Each of these five items was evaluated as present (yes/no). The Trauma Team Performance Observation Tool (TPOT) was used by Capella.^7^ Trained evaluators assessed team performance by the following categories: leadership, situation monitoring, mutual support, and communication skills. Each of these categories was evaluated using a set of questions. For example, under the leadership category was the question: “Does the leader continually render the plan of care to the team and ensure task prioritization?” and this was measured on a five-point Likert scale.^7^ The Trauma NOTECHS is a five-point Likert scale with four domains: cooperation and resource management, communication and interaction, assessment and decision-making, and situation awareness/coping with stress. The scale was modified in Steinemann et al. by a panel with extensive trauma experience to make it more relevant to trauma. Teamwork was rated by critical care nurses and trained medical students.^8^ In Holcomb’s study, senior physicians and nurses used the Trauma Team Evaluation Tool to evaluate team organization on a 0–2 scale. Teams were more effective if there was a clearly defined team leader with other team members assuming functional roles.^16^

Four scales evaluated leadership in isolation rather than in the context of teamwork.^9^, ^13^, ^15^, ^17^ The modified Campbell Leadership Descriptor Survey tool was used in Sakran’s study and was filled out by team members (e.g. residents, trauma fellows, and nurses) after a resuscitation. The evaluation tool is rated on a four-point Likert-type scale in nine leadership domains: vision, management, empowerment, diplomacy, feedback, innovative/creative, style, energy, and leadership.^18^ The LBDQ was the only scale used in more than one study.^9^, ^13^ The LBDQ, which was developed at Ohio State University in the 1950s for team members to describe leadership behavior, evaluates leaders in two behavioral domains: initiating structure and consideration. Initiating structure includes task-oriented behaviors and consideration includes people-oriented behaviors. Trained observers evaluate how much the leader displays these behaviors by marking always, often, occasionally, seldom, or never. There are 40 total behaviors including the following: lets group members know what is expected of them, maintains definite standards of performance, and treats all group members as his/her equals.^19^ Cooper et al. first used this scale in medical resuscitation. The authors eliminated consideration items because these behaviors were unlikely to be important under time constraints, and the 10 initiating structure items were adapted to fit resuscitation scenarios. In the end the authors claimed the modified LBDQ had excellent unidimensional validity.^9^ In Yeung et al. they used Cooper’s modified LBDQ to evaluate leadership behavior and also showed high inter-rater reliability.^13^

### 3. How should we train leaders?

All resuscitation leadership training programs used a combination of didactic teaching with simulations and showed improvements in team performance. However, the length, intensity, and experience level of participants varied widely.

Seven studies included in our review evaluated and described methods for training physicians to become better leaders. Training programs included didactic teaching to emphasize key leadership behaviors followed by live, mannequin, or computer-based simulations to practice and solidify new skills/behaviors, and all programs demonstrated improved team performance. Programs differed in the length of instruction and the training level of participants. Few programs focused on other methods of training such as textbook, small group, apprenticeship, or panel discussions.^7^, ^8^, ^12^, ^16^, ^20^, ^21^ In Fernandez et al., 231 medical students and residents used a computer-based simulation training module targeting appropriate resuscitation teamwork behaviors including goal development, strategy formulation, communication, and leadership. During follow-up high-fidelity simulation, trainees demonstrated improved leadership behavior scores as well as patient care scores determined by items such as appropriate chest compressions.^21^ In Steinemann et al., residents and attending physicians participated in a team-based training program consisting of videotaping and debriefing mannequin simulations. After training, 100 trauma resuscitations were recorded and analyzed, and participants in the training program had improved task performance and achieved decreased emergency department resuscitation time.^8^ In Hunziker’s leadership training, participants were taught to perform four practical items: 1) decide what to do; 2) tell your colleagues what to do; 3) make short and clear statements; 4) adhere to a treatment algorithm. Students subsequently had better outcome measures including time to beginning cardiopulmonary resuscitation (CPR) and maintaining the correct chest compression rate.^11^

Two studies used standardized training programs rather than developing their own. One used TeamSTEPPS developed by the U.S. Agency for Health Care and Quality and the other used Standardized Trauma and Resuscitation Team Training (STARTT) program developed by the UK National Health Service.^7^,^20^ TeamSTEPPS is widely used by both the defense and airline industries and has been used in healthcare settings to train leaders.^7^ The program consists of a didactic course with text and supporting DVD supplemented by video vignettes to illustrate key concepts. After training, teams using this program had improved scores in leadership, situation monitoring, mutual support, and communication as measured by the Trauma Team Performance Observation Tool. Additionally, teams had faster patient arrival rates to the CT scanner, endotracheal intubation, and operating room in live trauma resuscitations after receiving leadership training.^7^ The STARTT program trains trauma team members by incorporating a seven-chapter textbook, lectures, and discussion of key performance goals including leadership, communication, and situational awareness followed by high-fidelity trauma simulation as well as live-actor simulation.^20^ After training, participants in Zeismann’s study had high satisfaction and significant improvement in attitudes towards teamwork.^20^

While programs did not differ widely in method of delivering content, they varied dramatically in length and intensity as well as the level of experience of trainees. Despite these differences, they all demonstrated improved leadership and team functioning after training. The shortest training programs had didactic sessions that were under a half hour. Hunziker et al. showed technical improvements in delivering CPR after just 10 minutes of leadership training, and students demonstrated sustained improvement after a four-month follow up.^11^ Fernandez’s 25-minute computer-based training module showed improved team performance and could be easy to implement.^21^ The longest program was a 28-day military training course using human patient simulation (HPS) and included hands-on clinical experience, case reviews, lectures, skill station sessions, and a before-and-after test of didactic trauma knowledge.^16^ Team training was emphasized throughout the 28-day course but was not the only topic addressed. Human patient simulation was an effective teaching and evaluation tool, and the 10 teams undergoing training had team performance scores nearing expert teams after 28 days.^16^ The other programs ranged in length from two hours to eight hours.^7^, ^8^, ^12^, ^13^, ^20^ In addition, trainees varied by program including medical students, residents, and attendings in various combinations. Therefore, training programs could not be compared based on the level of experience of participants even though each group is likely to require tailored training by role. No studies commented on whether training program content was changed, altered, or designed specifically for level of expertise, and program content was not available to analyze any meaningful difference in course complexity.

## DISCUSSION

According to the Centers for Disease Control and Prevention, unintentional injury remains the leading cause of death in people under 44 years of age and the fifth overall cause of death in the United States.^22^ In addition, traumatic injury has grave economic consequences with $80.2 billion in medical costs and $326 billion in lost productivity annually.^23^ While the development of regional trauma centers had a rapid and profound impact on trauma care and outcomes, progress has slowed in recent years, and new strategies are needed to improved trauma outcomes. In this review, we find that leadership and teamwork have a significant impact on trauma processes of care. Encouragingly, we found that leadership can be improved with dedicated training in as little as 10 minutes.

Leadership is a multi-dimensional, complex behavior that includes effective communication, efficiency, decision-making, and resource management skills. As a consequence, measuring leadership is challenging. Compared with other industries, the importance of leadership in trauma care has only recently been recognized.^1^ Most leadership measurement tools have been borrowed from other industries and adapted to trauma rather than being developed specifically for trauma care. Our review found that only one tool, the modified LBDQ, was used in more than one study of leadership during resuscitations. In general, most leadership measurement tools have not been subject to rigorous psychometric validation. Moreover, raters included individuals with varying levels of medical sophistication (ranging from medical students to experienced trauma surgeons). It is impractical for senior physicians or nurses to serve as raters for most trauma resuscitations. Future efforts should focus on developing a tool that can be used by raters with limited medical training while maintaining high reliability and validity.

The optimal style of leadership is affected by patient characteristics and team composition. ISS and team experience determine which leadership style is optimal in trauma. Directive leadership was more effective with high ISS and inexperienced teams, and empowering leadership was more effective with lower ISS and more experienced teams. When a patient is more severely injured, decisions must be made quickly with limited time for thorough discussion, thereby favoring a directive approach. Similarly, when team members are relatively inexperienced decision-making should default to an experienced leader who can direct subordinates. Empowering leadership is more effective with experienced team members who already possess the knowledge to make their own decisions, as the leader is free to oversee and guide the resuscitation. In addition, empowering leadership facilitates learning for team members by allowing them to make their own decisions and debate these with senior clinicians. 17 In many cases there is only time for this type of deliberate discussion when patients are less severely injured. Therefore, for critical patients who require physicians to deliver swift and decisive care, conflict exists between which leadership style is best for the patient and which is best for training physicians. More research is necessary to determine which style of leadership should be used in training facilities to provide the best education to residents while simultaneously providing quality care for patients.

With the development of emergency medicine as a recognized medical specialty, the composition of many trauma teams changed. Emergency and trauma physicians began jointly running trauma cases, and cross-disciplinary decision-making became increasingly common. In this structure, emergency and trauma physicians collaborate in order to decide on a unified action plan. In contrast, parallel decision-making occurs when emergency physicians and trauma surgeons make their own decisions without consulting one another. Our results showed that if emergency physicians and surgeons share leadership roles by collaborating (cross-disciplinary) care is delivered faster if each physician makes decisions independently (parallel decision-making). See SCD 1. Overall, the leadership style, structure, and approach will not be the same in all circumstances, and optimal leaders will need to be adaptable in order to apply the correct style and structure of leadership to situation.

This review finds compelling evidence that leadership is a skill that can be taught and improved upon. There are many possible educational modalities including lectures, textbooks, small groups, and workshops that could be used to train leaders. However, the optimal training method and duration is elusive. In our review, the most commonly used training method was a combination of didactics and simulation. While this strategy was effective, simulation training, especially with the use of high-fidelity mannequins, is resource intense and may be cost prohibitive. Training programs also varied in length and trainee level of experience. Training programs included medical students, residents, and attending physicians in many combinations. Therefore, while medical students and physicians likely require different training, we could not look for changes in design, length, or content based on trainee level of experience. Future research is needed focusing on each group individually to develop the most cost effective and efficient training methods.

Optimizing outcomes for trauma victims requires a multi-dimensional approach. Common strategies in the past have included new surgical techniques, new drugs, and new trauma center systems. Improving leadership/teamwork may be a novel mechanism to accelerate progress in the future. Our review finds that leadership is associated with improvements in processes of care during resuscitations and is highly trainable. However, the literature to date is limited by the lack of 1) patient level outcomes 2) easy-to-use, validated measurement tools, and 3) understanding of the optimal training methods. To move forward, easy-to-use, validated tools and cost-effective training programs must be developed. Future efforts should focus on confirming that leadership not only affects processes of care but also that this translates directly to improved patient outcomes.

## LIMITATIONS AND CONCLUSION

There were several limitations in this study. We only searched PubMed, which is typical for medical research, but since this topic spans sociology and organizational theory we may have missed relevant articles. We think this is unlikely given experts in trauma and emergency medicine performed an ancestral review by tracking down references cited by relevant sources to capture any remaining articles that fit our criteria. There is a limited pool of studies that focused solely on trauma. To expand the number of studies we decided to include medical resuscitation because it also requires a team to act in a time-sensitive manner to provide the best possible patient care. Importantly, the key findings of the review are unchanged if we restrict the analysis to only studies of trauma resuscitation. Since no gold standard currently exists, each study used its own tool to evaluate leadership and teamwork. Most scales were only partially validated, and only the LBDQ was used in more than one study. The variety of measurement tools made it impossible to pool together results. Additionally, most studies used process-of-care improvements as endpoints rather than patient outcome. Past studies have shown that this assumption is solid, but studies that look at patient outcome directly are necessary to confirm these results.^24^, ^25^

Despite these limitations, addressing team organization and leadership skills results in improved speed and quality of care for patients in resuscitation settings. Therefore, leadership and teamwork training could be a key issue for improving patient care, but our level of knowledge about what defines and how to measure effective teamwork/leadership is lacking. Future efforts should focus on better defining, teaching, and assessing leadership and trauma team organization and definitively equating improvements in processes of care with improved patient outcomes.

## Figures and Tables

**Table 1:** Summary of trauma resuscitation studies on teamwork and leadership.

No	Setting	Design	Intervention	Sample size	Endpoints	Main results	Reference
1	Trauma Center	Retrospective observational study (video recordings)	none	425 trauma cases	Leadership quality Respect of ATLS standards	Evidence of leadership increases the level of adherence to ATLS standards of care	Hoff 1997 [6]
2	Trauma Center	Retrospective observational study (video recordings)	none	50 trauma cases	Video recordings to assess team work	Video recording is a good tool to assess how trauma resuscitations work, but is frequently not used adequately	Ritchie 1999 [^27^]
3	Training Center	Prospective observational study (pre-post observation)	28-day military trauma team training	30 team members 2 trauma cases	Trauma Team Evaluation Tool (TTET, team organization & expected tasks)	Team training was associated with significant improvement in team organization and TTET scores.	Holcomb 2002 [17]
4	Trauma Center	Prospective observational study	none	unknown	Leadership effectiveness measured by adapted Pearce & Sims scale Trauma team level of experience Trauma severity	Effectiveness of leadership depends on the severity of the patient’s injury and on the level of experience of the team Direct leadership is more effective when the patient’s condition is more severe, or when the team is less experienced Empowered leadership is more effective in less severe cases or when used with experienced teams	Yun 2005 [15]
5	Trauma Center	Retrospective observational study (video recordings)	none	387 trauma cases	Locally developed score of team performance	Errors in team organization lead to more errors in expected tasks	Lubbert 2009 [7]
6	Trauma Center	Prospective observational study (pre-post observation)	Team training using the Team STEPPS program and simulation	33 pre-training trauma cases 40 post-training trauma cases	Trauma Team Performance Observation Tool (TPOT) Delays to accomplish tasks related to ATLS standards of care	Training was associated with a better TPOT score Training was associated with a significant reduction of delays to accomplish ATLS-related tasks	Capella 2010 [8]

*ATLS, advanced trauma life support; TeamSTEPPS,* Team Strategies and Tools to Enhance Performance and Patient Safety

**Table 2:** Summary of live or experimental (simulation) studies on team work and leadership in medical resuscitation.

No	Setting	Design	Intervention	Sample size	Endpoints	Main results	Reference
7	Trauma Center (n=2)	Retrospective observational study (video recordings)	None	268 trauma cases	Structure of leadership Decision-making agreement ATLS expected tasks	Intra-disciplinary and cross-disciplinary shared leadership were better than solo or parallel decision making	Sarcevic 2011 [16]
8	Trauma Center	Prospective observational study (pre-post observation)	Team training using a 4-hour simulation session	137 trauma team members 141 pre-training trauma cases 103 post-training trauma cases	T-NOTECHS scale Delays to accomplish expected tasks	Team training was associated with an improved score of team work (T-NOTECHS) and reduced delays for expected tasks	Steinemann 2012 [9]
9	Trauma Center	Prospective observational study	none	81 trauma team members 22 trauma cases	Modified Campbell Leadership Descriptor Survey tool (CLDS) Delays to accomplish ATLS-related standards of care	A high leadership quality (CLDS score) was associated with a significant reduction in delays to accomplish ATLS-related expected tasks	Sakran 2012 [19]
10	General Hospital (Canada)	Qualitative study (Pre-post interviews)	Training of trauma team providers using the STARTT program	41 trauma team members	Providers’ attitudes toward Crew Resource Management’s (CRM) importance and simulation training	STARTT training was highly valued (high level of providers’ satisfaction) STARTT training was associated with an improved appreciation of simulation and CRM team work	Ziesmann 2013 [21]

*ATLS,* advanced trauma life support; *T-NOTECHS,* nontechnical skills scale for trauma
